# ANMM4CBR: a case-based reasoning method for gene expression data classification

**DOI:** 10.1186/1748-7188-5-14

**Published:** 2010-01-06

**Authors:** Bangpeng Yao, Shao Li

**Affiliations:** 1 MOE Key Laboratory of Bioinformatics and Bioinformatics Division, TNLIST/Department of Automation, Tsinghua University, Beijing 100084, PR China

## Abstract

**Background:**

Accurate classification of microarray data is critical for successful clinical diagnosis and treatment. The "curse of dimensionality" problem and noise in the data, however, undermines the performance of many algorithms.

**Method:**

In order to obtain a robust classifier, a novel Additive Nonparametric Margin Maximum for Case-Based Reasoning (ANMM4CBR) method is proposed in this article. ANMM4CBR employs a case-based reasoning (CBR) method for classification. CBR is a suitable paradigm for microarray analysis, where the rules that define the domain knowledge are difficult to obtain because usually only a small number of training samples are available. Moreover, in order to select the most informative genes, we propose to perform feature selection via additively optimizing a nonparametric margin maximum criterion, which is defined based on gene pre-selection and sample clustering. Our feature selection method is very robust to noise in the data.

**Results:**

The effectiveness of our method is demonstrated on both simulated and real data sets. We show that the ANMM4CBR method performs better than some state-of-the-art methods such as support vector machine (SVM) and *k *nearest neighbor (*k*NN), especially when the data contains a high level of noise.

**Availability:**

The source code is attached as an additional file of this paper.

## Background

Recently gene microarray technology has become a fundamental tool in biomedical research, enabling us to simultaneously observe the expression of thousands of genes on the transcriptional level. Two typical problems that researches want to solve using microarray data are: (1) discovering informative genes for classification based on different cell-types or diseases [1]; (2) clustering and arranging genes according to their similarity in expression patterns [2]. Here we focus on the former, especially on microarray classification using gene expression data, which has attracted extensive attentions in the last few years. It is believed that gene expression profiling could be a precise and systematic approach for cancer diagnosis and clinical-outcome prediction [3].

With about ten years of research, many algorithms have been applied to microarray classification, such as nearest neighbor (NN) [4], artificial neural networks [5], boosting [6], support vector machine (SVM) [7], etc. Many commonly used classifiers are rule-based or statistical-based. One challenge of these methods on microarray data is the small sample size problem. With the limited number of training samples, it is difficult to obtain domain knowledge for rule-based systems or get accurate parameters (such as mean value and standard deviation) for statistical-based approaches.

Other than adopting rule-based or statistical-based classification methods, in this paper we use a case-based reasoning (CBR) [8] approach to design a robust microarray classifier. CBR usually requires much less domain knowledge than rule-based or statistical-based systems, because it does not heavily rely on the statistical assumptions on the data during the classification procedure. It maintains a case-base of previous problems and their solutions, and solves new problems by reference to this case-base. NN can be viewed as the simplest form of CBR methods. With a complicated comparative study, in [9] it was concluded that NN performed better compared with more sophisticated ones. Moreover, [10] observed that CBR is particularly useful for applications in life sciences, where we lack sufficient knowledge either for formal representation or for parameter estimation. [11] reviewed previous research works in applying CBR to bioinformatics domains. In the problem of microarray classification, however, except the simplest form NN, CBR classifiers were considered in only a few literatures [11,12] and was only tested on some simple data sets.

In order to design an effective classifier, dimension of the microarray data should be reduced. Of the thousands of genes in a microarray data, only a small fraction are informative from the aspect of biological meaning or classification performance [13]. In this work we propose a novel additive nonparametric margin maximum (ANMM) method for feature selection. Three properties determine ANMM's superiority in feature selection for microarray data: (1) ANMM is a nonparametric method which requires less restrictive assumptions about the original data, and thus is suitable for dealing with microarray data [14]. (2) The feature reduction criterion for ANMM is defined based on gene pre-selection and sample clustering, which renders ANMM insensitive to outliers or mislabeled samples. (3) There exist some relationships between ANMM and CBR, and therefore the performance of CBR classification can be improved by ANMM feature selection.

Using ANMM for feature selection and CBR for classification, a novel ANMM4CBR method is established in this paper. The performance of ANMM4CBR is tested on one simulated data and four publicly available data sets, comparing with some well-known methods including SVM, *k*NN and LogitBoost, as well as the other CBR methods that have been applied to microarray classification. We show that ANMM4CBR can result in exciting classification results, especially on the data which contains a high level of noise.

## Methods

### Overview of ANMM4CBR

In a microarray data classification problem, we are given *N *training samples , where *x*_*i *_is an *M*-dimensional vector in the feature space and *y*_*i *_∈ {0, ⋯ *K *- 1} is the class label. The set of samples in the *k*th class are denoted as *ω*_*k*_, *i.e. x*_*i *_∈ *ω*_*k *_means *y*_*i *_= *k*. The genes are denoted as , where *ϕ*_*m *_(*x*) is the expression value of sample *x *on the *m*th gene. The learning task is to select a subset from all the genes, and define a similarity measurement based on the selected genes. When given an unlabeled sample, we expect to predict the category of this sample using the selected genes and the defined similarity measure.

In this paper, we propose a CBR-based method to construct the classifier. CBR classifiers use a philosophy that plays a vital role in human decision making. They try to solve new problems by retrieving previously solved cases from a case-base. The process of solving new cases contributes new information to the system, and this new information can be used for solving other future cases. In [15], CBR method is described in terms of four phases. In the first phase, CBR *retrieves *old cases similar to the new one. The second phase *reuses *the solutions of the retrieved cases for solving the new case. The third phase *revises *the solution, e.g. by a human. Finally, the fourth phase *retains *the useful information which is obtained when solving this case.

**Figure 1:**
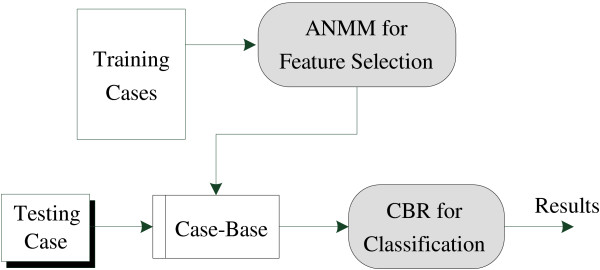
**Framework of ANMM4CBR for microarray classification**. ANMM4CBR contains two modules, ANMM for feature selection and CBR for classification. Both ANMM and CBR are suitable for dealing with microarray data, which usually contain noisy information and only a small number of training samples are available.

Here we focus on the *retrieving *and *reusing *phases, and propose a novel ANMM4CBR method for classification (see Figure 1). For feature selection, we develop a novel ANMM method, which additively optimizes a nonparametric margin maximum criterion. We define this criterion based on gene pre-selection and sample clustering to make it robust to noise and outliers. In our CBR classifier, each class contains one case-base. For a testing case, we retrieve similar cases from each case-base, and combine the results of all the case-bases to provide a classification label.

According to the notion of CBR, we can revise the prediction results of testing samples and then add them to the case-bases. The *revising *and *retaining *phases, however, are not the focus of this paper and will not be mentioned in the following descriptions. Details of the ANMM and CBR modules are described below.

### Additive Nonparametric Margin Maximum for Feature Selection

Here we introduce an ANMM feature selection method, which uses an additive method to optimize a nonparametric margin maximum (NMM) criterion. The NMM criterion is defined based on *nearest between-class distance maximization *and *furthest within-cluster distance minimization*. We first describe the NMM criterion, and then present the additive optimization method.

#### Nonparametric Margin Maximum (NMM) Criterion

The goal of feature selection is to identify *informative genes *from thousands of available genes. The informative genes are those that have high discriminative powers, and have low correlations between each pair of them [16]. Selecting informative genes helps not only overcome the curse of dimensionality problem and thus improve the prediction accuracy, but also reveal meaningful biological explanations of the dataset. Theoretically, any wrapper or filter feature selection method, such as t-test, mutual information measurement, etc, can be used. However, one drawback of these approaches is that the feature selection criterion is designed regardless of the classifier design. In [17], it has been observed that almost all feature selection methods have some assumptions of the distribution of the data, and these assumptions usually affect the performance of the classifiers. Therefore, it is important to design a feature selection method that is suitable for the classification method that will be used.

Bressan and Vitrià [17] showed that there is a close link between nonparametric discriminant analysis (NDA) [18] and instance-based classifiers. In that work, a modified NDA was applied to improve the performance of NN for face recognition. Since CBR-based methods also belong to instance-based classifiers, we believe that the idea of NDA also helps to improve, at least not downgrade the performance of CBR. Our NMM criterion is defined based on the notion of NDA. Instead of directly using the furthest within-class distance as in the original NDA method, in our method training samples in each class *ω*_*k *_are firstly grouped into many clusters {} so that the samples in each cluster have similar patterns. The objective of NMM is to maximize the between-class distance of samples while minimize the within-cluster distance. For one sample *x*_*i *_∈ *ω*_*k*_, we define its nearest between-class neighbor as(1)

Similarly, its furthest within-cluster neighbor is defined as(2)

where *C *[*x*] indicates the cluster that *x *belongs to.

Then the nonparametric margin of *x*_*i *_is(3)

where  is the nonparametric nearest between-class distance for *x*_*i*_, and  is the furthest within-cluster distance. Obviously, the larger Θ_*i *_is, the more likely that *x*_*i *_is correctly classified. Therefore the learning objective of NMM is to select a subset of genes  from Φ to maximize the nonparametric margin for all the samples, *i.e*. to maximize(4)

where *δ*_*i *_is the sample *x*_*i *_in the space of selected features, which is represented as *δ*_*i *_= [*h*_1_(*x*_*i*_), ⋯, *h*_*T*_(*x*_*i*_)]^*T*^.

Not surprisingly, we find that if each class contains only one cluster, the NMM criterion is equal to the optimization objective of NDA (see proof 1). Since it has been proved that there are close relationships between NDA and instance-based classifiers such as NN [17], we believe that our margin maximum criterion also benefits the design of a robust CBR classifier. Moreover we replaced the furthest within-class distance with furthest within-cluster distance, which makes our approach more robust to outliers, considering that the outliers that usually exist in microarray data might make the furthest within-class distance extremely large. Another major difference between our method and NDA is that, NDA performs feature reduction by finding a weighted combination of all the features, while NMM aims at selecting a subset of features. This property is important since the selected features can be used to reveal some biological significance.

##### Proof 1

The Nonparametric Margin Maximum (NMM) criterion in Equation (4) can be expanded as the following(5)

When each class contains only one cluster, we have(6)

where *S*^*B *^and *S*^*W *^are between-class and within-class scatter matrix for NDA respectively. Therefore we can conclude that when each class contains only one cluster,(7)

where the left-hand side is the NMM criterion and the right-hand side is the NDA optimization criterion.   □

#### Feature Pre-selection and Clustering

In our method, we normalize the original data and then perform feature pre-selection and sample clustering to define within-cluster neighbors. We use the same normalization method as in [19], which includes base 10 log-transformation as well as normalization to mean 0 and variance 1. For the data that contains negative values, we do not perform log-transformation.

In microarray data, the gene dimension is extremely large compared to the small number of samples. Many of these genes are not differentially expressed across the samples of different classes and thus do not contain very useful information. It is likely that too many non-informative genes in the data will undermine the clustering results. In order to improve the clustering performance, we implement gene pre-selection before clustering. Another benefit of removing some non-informative genes is that it can drastically ease the computational burden in subsequent processing procedures.

Approaches that can remove non-informative genes have been studied in many literatures, for instance t-test [20], mutual information (MI) maximization [16], etc. Instead of these parametric methods, we use a nonparametric scoring algorithm presented in [13]. For binary classification which involves two classes *ω*_0 _and *ω*_1_, the score of a feature *ϕ*_*m *_is(8)

where ⟦A⟧ equals 1 if A is true, otherwise 0. |*ω*| is the number of samples in *ω*.

The genes whose scores are below a threshold *θ*_*p *_will be removed, and the remaining genes are used for further processing. Compared with the parametric methods such as t-test and MI maximization, this method is less sensitive to outliers, since it does not rely on any statistical values (mean, standard deviation, etc.) of the data, which can be highly affected by outliers.

This nonparametric method can be easily generalized to multiclass problems by considering all the possible binary cases. For a *K *class problem, the score of a feature *ϕ*_*m *_is(9)

After gene pre-selection, we group samples in each class into some clusters. Although there are many choices of clustering approaches, hierarchical clustering [21] is the most commonly used one for microarray analysis. The preference of hierarchical clustering in microarray analysis is due to its good performance [2] and, moreover, it does not require a pre-specification of the number of clusters.

We use the most common type of hierarchical clustering. At the initial level, each sample forms its own cluster. At each subsequent level, the two 'nearest' clusters are combined to form one bigger cluster. We use *method = 'furthest' *which means the distance between two clusters is the maximum of all the distances between any sample in one cluster and any sample in the other cluster. The 'furthest' metric is used since it is not highly sensitive to outliers compared with the other metrics such as 'nearest' and 'average'. We empirically set a threshold *θ*_*h *_for clustering, which means that for each class, the clustering procedure terminates when the distance between any two clusters is larger than *θ*_*h*_.

#### Additive optimization method

Here the NMM criterion is optimized in an additive approach, which operates iteratively. At each iteration, one feature is selected. Assuming that until the (*t - *1)-th iteration the margin is *J*_*t*-1_, at iteration *t *the feature *h*_*t *_will be selected to maximize(10)

During the optimization procedure, however, when one feature is selected, for each sample its nearest between-class neighbor and furthest within-cluster neighbor might change. In another word, the optimization of *J*_*t *_might change *J*_*t*-1_, and for each sample, many other samples might become its nearest between-class neighbor or furthest within-cluster neighbor in subsequent processing. Therefore we should maintain the distance between any two samples in each iteration, which is computationally expensive. In order to reduce computational complexity, we maximize the following formula instead of directly optimizing Equation (4),(11)

Proof 2 shows that Equation (11) is a low bound of Equation (4), which implies that we can maximize Equation (4) by optimizing Equation (11).

##### Proof 2

**Figure 2:**
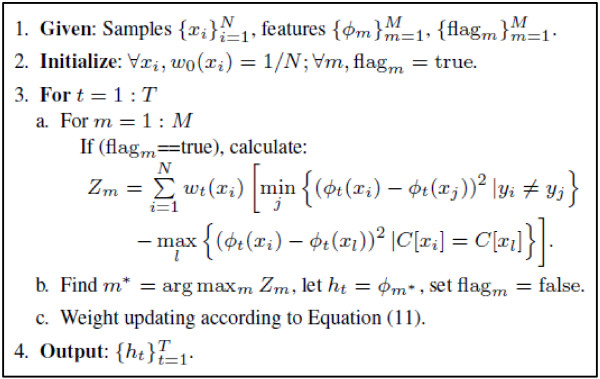
**Additive optimization of the NNM criterion**. flag_*m *_indicates whether *ϕ*_*m *_has been selected. It is true if *ϕ*_*m *_has been selected, otherwise false.

With the criterion of Equation (11), at each iteration we can independently treat each feature to select the best one, regardless of the features that have been selected at previous iterations. This implies that we can test each feature on training set and select the top-ranked ones. However, [16] has observed that simply combining the top-ranked genes often does not form a good feature set. One reason is that the top-ranked genes could be highly correlated, and therefore the selected features might contain much redundant information. In order to overcome this problem, similar in the way that the boosting method [22] does, we assign weights  to training samples. Initially all samples share the same weight. When one feature is selected, the weights are updated with the principle that the sample that has a larger margin will get a lower weight, and vice versa. The weights of the samples are updated by(13)

where , and *α *is a positive parameter. Algorithm flow of the additive optimization method is shown in Figure 2.

### Case-Based Reasoning Classifier

Rather than using the traditional CBR methods in which all the samples form a single case-base, here we treat samples in each class one case-base. For a *K*-class problem, there are *K *case-bases . Given an input sample *x*, ANMM4CBR retrieves several similar cases from each case-base. The distance between *x *and a sample *x' *in case-base is measured by(14)

If there are *l*_*k *_samples in the case-base *ω*_*k*_, *β*·*l*_*k *_cases that have small distances from *x *will be selected as similar cases, where *β *is a parameter that controls the number of samples that will be retrieved from each case-base. The distance between *x *and *ω*_*k*_, *D*(*x*, *ω*_*k*_) is the average of the retrieved *β*·*l*_*k *_distances. In the ANMM4CBR method, we calculate the distance from *x *to each case-base *ω*_*k*_, and *x *belongs to the class which relates to the minimum distance *D*(*x*, *ω*_*k*_).

## Results and Discussion

We carried out experiments using simulated data as well as real microarray data to test the performance of ANMM4CBR. There are four parameters to be chosen in ANMM4CBR: gene pre-selection threshold *θ*_*p*_, cluster-stopping threshold *θ*_*h*_, weight-updating parameter *α*, and the parameter *β *for case retrieve. We empirically set *θ*_*p *_and *β *to 0.7 and 0.3 respectively, which means the genes with score values smaller than 0.7 will be removed in the gene pre-selection procedure, and CBR will retrieve 0.3|*ω*| cases from a case-base which contains |*ω*| cases. The other two parameters, *θ*_*h *_and *α*, are data-dependent. Therefore we adopted cross-validation to choose them. After the whole data were split into training and testing sets, we used five-fold cross-validation on training set to evaluate the performance of ANMM4CBR with different values of *θ*_*h *_and *α*. Then the best combination of *θ*_*h *_and *α *was selected to train an ANMM4CBR classifier using all training samples. The tuning parameters for *θ*_*h *_are 0.8, 0.9, ⋯, 1.5, and for *α *are 0.3, 0.4, ⋯, 1.0. Please see additional file 1 for the source code of the ANMM4CBR method.

### Simulation

We first consider simulated data. We used a noisy version of the simulated data in [23]. The original data assumes three different normal distributions for both insignificant genes (null cases) and significant genes. There are 72 samples (47 positive and 25 negative) in the dataset, and out of 1000 genes there are 10 significantly differentially expressed ones. Please refer to [23] for more details of this data.

We compared ANMM4CBR with several typical classification methods, including support vector machine (SVM) [7] with linear kernal, *k*-nearest neighbor (*k*NN, we set *k *= 3), and LogitBoost [6]. In the above three algorithms, only LogitBoost is a combination of feature selection and classification. There should be feature selection methods for SVM and *k*NN classification. Here two feature selection methods were tested. One is the Between-group to Within-group (BW) ratio method described in [9]. The BW ratio for gene *m *is(15)

where *x*(*m*) and *x*_*k*_(*m*) denote the average expression value of gene *m *across all samples and across samples that only belong to class *k *respectively. *x*_*i*, *m *_is the expression value of gene *m *in the *i*th sample. ⟦·⟧ is the indicator function which has been described in Equation (8). Another feature selection method we used is the Minimum Redundancy - Maximum Relevance (MRMR) method proposed in [16], which has been proved very effective for microarray data analysis. Other than simply picking the top-ranked genes, MRMR also minimizes redundant information in the selected genes by measuring correlations between different genes. We used the FCQ criterion to optimize MRMR, which means using F-test to compute the maximum relevance *V*_*F *_and using Pearson correlation coefficient to compute the minimum redundancy *W*_*c*_, and combining them with their quotient, max(*V*_*F*_/*W*_*c*_).

The simulated data was randomly and equally divided into three parts, of which two parts were used for training and the third part was used for testing. In each experiment we constructed a noisy training data by assigning a randomly chosen, incorrect label to 20% of the training samples. We use noisy data because we want to test the performance of the algorithms confronting noises, which is usually the case for real microarray data. Another reason for the usage of noisy data is, we found that if there is no noise in training data, all algorithms used in this paper can get a 100% testing accuracy if we choose appropriate number of features. We used the noisy training samples to train classifiers and the test error rates were computed by testing samples. In order to obtain more replicable results [24], we repeated this procedure for 100 times. Here we also investigated the performance of ANMM4CBR method without feature pre-selection and sample clustering.

**Figure 3:**
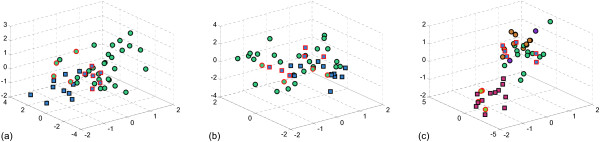
**Visualization of training samples using top 3 selected features by different feature selection methods**. The feature selection methods are: (a) BW, (b) ANMM without feature pre-selection and sample clustering, (c) ANMM. Results of MRMR were not listed due to space limitation. Figure 4 shows that MRMR did not perform better than BW on this data. In these figures, different marker types represent samples in different classes, and the mis-specifications are depicted with red edge. In (c) samples in different clusters are filled with different colors.

Figure 3 shows the distribution of training samples with top 3 selected features by different feature selection methods. We can see that the BW method cannot well separate the two classes, since the mis-specifications made the data not separable by the BW criterion. In the ANMM method, samples in each class were clustered into many groups, which is illustrated in Figure 3(c). We can see that the mis-specifications were clustered into different groups with the other samples, so that they did not exert great influence to the feature selection procedure. Figure 3(c) shows that the training samples of different classes were well separated, excluding the mis-specifications. The ANMM result without feature pre-selection and sample clustering are listed in Figure 3(b). The result in Figure 3(b) is even worse than that obtained by BW, which shows that feature pre-selection and sample clustering can really improve the performance of ANMM in noisy data.

**Figure 4:**
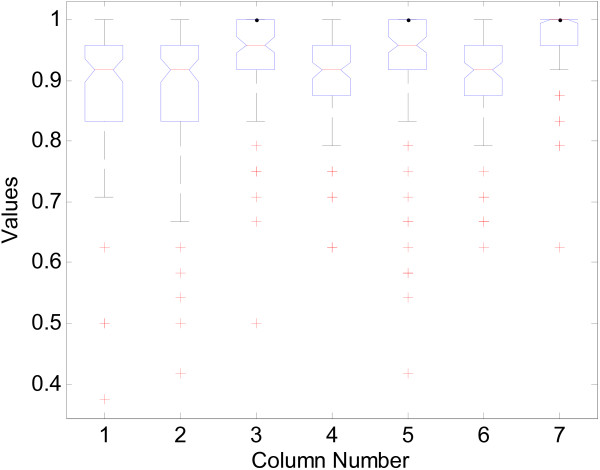
**Boxplots of the accuracy on simulated data**. "Values" indicate the accuracy. Each column indicates different algorithms: 1 - BW+*k*NN; 2 - MRMR +*k*NN; 3 - BW+SVM; 4 - MRMR+SVM; 5 - LogitBoost; 6 - ANMM4CBR without feature pre-selection and sample clustering; 7 - ANMM4CBR.

Boxplots of the accuracy on various methods are shown in Figure 4. For each method, the feature number was chosen by minimizing the average error rates. We can see that ANMM4CBR resulted in much higher accuracy. If we do not add noise on training data, all approaches can get 100% testing accuracy. This shows that ANMM4CBR is very robust when dealing with noisy data, while the performance of the other methods will be undermined because of the noise in training samples.

### Real Data

#### Data sets and experimental set up

In this section we carry out experiments on four publicly available real data sets that have been widely studied. Brief descriptions of these data sets are as follows. Please refer to the original papers for more details of each data set.

##### Leukemia

This data comes from a study [3] of 72 leukemia patients using Affymetrix HuGeneFL array. It contains 47 cases of acute lymphoblastic leukemia (ALL) and 25 cases of acute myeloid leukemia (AML) with the expression levels of 7,129 genes.

##### Colon

The Colon data contains expression levels of 40 tumor and 22 normal colon tissues. The data was analyzed with an Affymetrix oligonucleotide array complementary to more than 6,500 human genes. We used 2,000 genes with the highest minimal intensity across the samples selected by [25].

##### SRBCT

The SRBCT data [5] contains gene-expression data from cDNA microarrays of 2308 genes. The 63 samples include four subtypes of small, round blue cell tumors of childhood, which are 12 neuroblastoma (NB), 20 rhabdomyosarcoma (RMS), 8 non-Hodgkin lymphoma (NHL), and 23 Ewing family of tumors (EWS).

##### GCM

GCM (Global Cancer Map) [26] is a very complicated data, which consists of 198 human tumor samples covering 14 different cancer types. The gene number is 16,063. Please refer to [26] for details of this data set.

The procedure of each experiment was implemented as that on the simulated data. Each data set was split into three parts, of which two parts for training and the left part for testing. For each method, this procedure was repeated for 100 times, and the averages and standard deviations of accuracy were taken for performance evaluation.

#### Results

Similar to that on simulated data, here we also compared ANMM4CBR with SVM, *k*NN and LogitBoost. BW and MRMR were used to select features for SVM and *k*NN classification. Since the standard SVM is tailored for binary classification, in multiclass data sets we used the one-versus-all (OVA) [26] approach, which firstly solves many binary problems and then combines the results to solve the multiclass problem. Given a *k *class problem, OVA trains *k *binary classifiers, each focuses on classifying one class against the others. A new sample will take the class label of the classifier with the largest real valued output from all *k *classifiers. For LogitBoost, we used the same approach of [6], in which multiclass problems were solved by combining OVA results in a Bayes framework.

Table 1 gives the classification results of the six methods on the four microarray data sets. The results demonstrate that these algorithms show different performance on different data sets. On Leukemia data, all methods achieve comparable results, with ANMM4CBR and MRMR+SVM perform slightly better. On Colon data, ANMM4CBR performs better than the other methods by a large margin. We can see that with different number of selected features, ANMM4CBR consistently achieves the highest accuracy. On SRBCT data, the results are different with different numbers of features. When the feature number is small, SVM and LogitBoost perform better than ANMM4CBR; when the feature number is large, ANMM4CBR performs better. Table 1 also shows that, results of ANMM4CBR on GCM are not encouraging. SVM performs better than the other algorithms on GCM data.

**Table 1:** Average classification accuracy and standard deviation.

# Iteration		10	20	30	40	50
Leukemia	BW+*k*NN	95.7 ± 1.2	96.9 ± 1.8	96.6 ± 2.2	96.6 ± 1.2	96.8 ± 1.7
	MRMR+*k*NN	**96.5 **± **2.5**	96.4 ± 2.1	97.4 ± 1.7	96.9 ± 2.2	95.8 ± 2.4
	BW+SVM	95.6 ± 1.3	95.7 ± 1.7	95.9 ± 2.2	96.2 ± 2.3	96.9 ± 1.2
	MRMR+SVM	96.4 ± 2.5	96.8 ± 3.6	**97.6 **± **2.0**	**97.1 **± **2.7**	96.8 ± 3.4
	LogitBoost	95.3 ± 2.9	96.0 ± 2.4	96.6 ± 1.8	96.6 ± 2.8	96.7 ± 1.7
	ANMM4CBR	96.3 ± 2.4	**97.5 **± **1.7**	97.3 ± 1.8	96.6 ± 1.7	**97.0 **± **2.3**

Colon	BW+*k*NN	81.2 ± 8.1	82.8 ± 7.5	83.5 ± 4.2	83.4 ± 5.3	83.6 ± 6.5
	MRMR+*k*NN	83.7 ± 4.3	83.6 ± 7.9	84.2 ± 6.0	83.8 ± 5.9	83.5 ± 6.9
	BW+SVM	84.0 ± 4.3	83.6 ± 6.4	83.6 ± 6.0	84.2 ± 7.2	84.5 ± 7.9
	MRMR+SVM	85.4 ± 5.8	84.1 ± 6.6	84.0 ± 4.0	84.6 ± 7.0	84.7 ± 8.1
	LogitBoost	84.4 ± 4.3	84.5 ± 8.9	83.6 ± 4.9	84.2 ± 6.8	84.1 ± 4.6
	ANMM4CBR	**86.3 **± **6.1**	**86.7 **± **5.6**	**86.2 **± **4.2**	**86.5 **± **5.6**	**85.6 **± **4.4**

SRBCT	BW+*k*NN (50)	94.4 ± 4.2	97.7 ± 2.1	97.9 ± 1.3	98.2 ± 1.6	98.0 ± 1.2
	MRMR+*k*NN (50)	78.4 ± 9.0	97.4 ± 1.9	98.6 ± 1.0	98.8 ± 0.9	98.2 ± 0.8
	BW+SVM (97)	94.0 ± 3.2	98.0 ± 1.4	98.4 ± 1.2	98.8 ± 0.9	99.2 ± 0.3
	MRMR+SVM (95)	81.0 ± 10.5	**98.2 **± **1.0**	**98.9 **± **1.3**	99.1 ± 0.7	99.2 ± 0.2
	LogitBoost (102)	**94.9 **± **3.1**	97.3 ± 1.8	98.0 ± 1.6	98.6 ± 1.1	98.6 ± 0.6
	ANMM4CBR (50)	90.3 ± 5.5	97.3 ± 1.5	98.8 ± 1.2	**99.3 **± **0.7**	**99.7 **± **0.3**

GCM	BW+*k*NN (50)	46.2 ± 4.7	47.4 ± 7.0	51.2 ± 4.9	52.6 ± 6.2	54.1 ± 5.8
	MRMR+*k*NN (50)	41.1 ± 7.1	42.7 ± 8.1	51.5 ± 1.6	58.3 ± 4.9	60.5 ± 5.9
	BW+SVM (254)	53.7 ± 5.1	58.1 ± 9.8	59.0 ± 6.6	**66.6 **± **6.7**	66.9 ± 3.6
	MRMR+SVM (259)	51.0 ± 7.7	**60.3 **± **7.0**	**61.8 **± **3.7**	64.8 ± 8.2	**67.8 **± **4.6**
	LogitBoost (273)	**57.1 **± **4.9**	60.1 ± 1.9	60.6 ± 4.0	62.1 ± 5.7	65.1 ± 5.4
	ANMM4CBR (50)	41.1 ± 1.2	51.0 ± 8.1	57.2 ± 6.9	61.1 ± 1.4	63.3 ± 3.9

Each experiment was carried out for 100 runs. The best results in different situations are labeled as black. Here the iteration number means the number of features used by each single classifier. In OVA case, the total number of genes may exceed the iteration number, since in OVA a multiclass problem is solved by considering many binary ones. In the parentheses we list the average number of features selected by each method when the iteration number is 50. See Table 2 for another experiment on multiclass data set.

We now take a closer look at the results in Table 1. We can see that ANMM4CBR performs much better than all the other algorithms on the Colon data, while only achieves comparative results on the Leukemia data. This is because Leukemia is a simple data on which many algorithms have reported impressive results. Therefore it is not surprising that all six algorithms in our experiment can have similar good results. In contrast, it was reported in [27] that the Colon data might have a sample contamination problem, and therefore the much better performance of ANMM4CBR on Colon data demonstrated its robustness to noise in the data sets.

Although when the feature number is 40 and 50, ANMM4CBR performs the best on SRBCT, on the two multiclass data sets ANMM4CBR cannot achieve comparative results with SVM and LogitBoost. It is shown in Table 1 that SVM and LogitBoost perform better than ANMM4CBR, and ANMM4CBR performs better than *k*NN. However, we argue that this does not indicate that ANMM4CBR cannot get good results on multiclass problems. Note that the same as *k*NN, ANMM4CBR can be directly used to solve a multiclass problem. Therefore in ANMM4CBR method the number of iterations is equal to the number of selected features. But in SVM and LogitBoost algorithms, we used OVA method to make the final prediction, which needs to solve *k *(class number) binary problems. When each binary classifier selects *s *features, the total number of selected features will be *O*(*s *× *c*). This means that with the same iteration number, SVM and LogitBoost have to use more features than ANMM4CBR and *k*NN.

Here we made another experiment on GCM. We compared ANMM4CBR with MRMR+SVM, which showed the best performance on GCM data in Table 1. In each comparison of this experiment, the number of features selected by ANMM4CBR was equal to the total number of genes that are selected for all the binary classifiers. Since we performed experiment for 100 times and in each time the total gene number may be different, we firstly carried out SVM experiment and then calculated the total number of genes. The results are shown in Table 2, which demonstrate that ANMM4CBR outperforms SVM by a large margin when they choose the same number of genes.

**Table 2:** Comparison of MRMR+SVM and ANMM4CBR on GCM data.

*s*/*T*	10/86	20/157	30/209	40/243	50/259
SVM+MRMR	51.0 ± 3.7	60.3 ± 4.0	61.8 ± 2.4	64.8 ± 4.5	67.8 ± 3.5
ANMM4CBR	**62.7 **± **4.8**	**66.1 **± **2.4**	**67.9 **± **3.5**	**69.1 **± **1.9**	**70.0 **± **2.9**

*s *is the number of genes in each binary SVM classifier, and *T *indicates the total number of different genes, *i.e*. the gene number for ANMM4CBR. In each situation the higher accuracy is labeled as black.

#### Compare with MOE4CBR

Since ANMM4CBR is a CBR-based method, we would like to compare it with other CBR methods that have been applied to microarray classification problems. Because both source code and data sets used in [11] are not available, we did not compare our method with the gene-CBR method in [11]. We compared ANMM4CBR with the mixture of experts for case-based reasoning (MOE4CBR) method [12], which builds CBR classifiers based on the idea of mixture of experts. We applied our ANMM4CBR method to the same microarray data with the same experimental set as that in [12], *i.e*., using the training and testing data suggested in [3] on the Leukemia data, and using leave-one-out cross-validation on the Lung data and average the results obtained from 20 trials. The Lung data contains 39 lung cancer samples with 18,117 gene expression levels. This data set is classified into two categories, recurrence (23 samples) and nonrecurrence (16 samples). The Lung data was not used in previous experiments because there are missing values. The same as that in [12], here missing values were imputed using the weighted *k*-nearest neighbor method [28].

In [12], the classification accuracies on Leukemia and Lung data are 74% and 70% respectively. 712 out of 7,129 genes were selected for Leukemia data classification and 1,811 out of 18,117 genes were selected for Lung data classification. When the same number of genes are selected, the classification results of ANMM4CBR are 91% on Leukemia and 75% on Lung. Moreover, on the Leukemia data, the best result obtained by ANMM4CBR is 94% when only 23 genes are selected. This shows that ANMM4CBR outperforms MOE4CBR, especially on the Leukemia data set.

## Conclusions

In the present work, we proposed a novel ANMM4CBR method for microarray classification. For feature selection, we proposed an ANMM method to additively optimize a nonparametric margin maximum criterion which was defined based on feature pre-selection and sample clustering. For classification, we adopted a CBR method, in which samples of each class form a case-base.

Some properties determine that the ANMM4CBR can be well applied to microarray data classification. (1) The nearest between-class distance maximum and furthest within-cluster distance criterion used in ANMM makes the feature selection less sensitive to noise or outliers existing in the data. (2) In classification phase ANMM4CBR uses a case-based reasoning method, which has been proved to be suitable for life science related problems [10]. (3) In microarray data the sample number is too small for us to estimate the accurate distribution of the data. In each step of ANMM4CBR (including feature pre-selection, clustering, feature selection, classification), we use nonparametric approaches which require less restrictive assumptions about the original data. (4) There are some links between ANMM feature selection and CBR classifier. Furthermore, ANMM4CBR can directly solve multiclass problems without having to convert them to many binary ones.

Our future research will focus on two directions. One is to study how to facilitate the parameters choice and gene number selection in ANMM4CBR. We have several parameters to tune, and it is time consuming to select a set of optimal parameters when dealing with a new data. Moreover in ANMM4CBR we should pre-specify the number of features to be selected. The other direction is to further investigate the relationship between ANMM and CBR, which was not theoretically warranted in this paper. We believe that a better algorithm can be designed by revealing the relationships between feature selection approach and the classifier.

## List of abbreviations

ANMM4CBR: additive nonparametric margin maximization for case-based reasoning; ANMM: additive nonparametric margin maximization; NMM: nonparametric margin maximization; CBR: case-based reasoning; SVM: support vector machine; NN: nearest neighbor; NDA: nonparametric discriminant analysis; MI: mutual information; BW: between-group to within-group; MRMR: minimum redundancy - maximum relevance.

## Competing interests

The authors declare that they have no competing interests.

## Authors' contributions

SL conceived and coordinated the research. BY designed the algorithms, carried out the experiments and drafted the manuscript. SL participated in the design of the experiments and helped to draft the manuscript. Both authors read and approved the final manuscript.

## Supplementary Material

Additional file 1 **We provide the source code and a readme file as an additional file.** The code was compiled with Visual Studio 2005. Click here for file
