# Supplementary material for: ANMM4CBR: a case-based reasoning method for gene expression data classification
Source: Algorithms Mol Biol. 2010 Jan 6;5:14. doi: 10.1186/1748-7188-5-14 (PMC2843690; doi:10.1186/1748-7188-5-14)
Supplement: Additional file 1 — We provide the source code and a readme file as an additional file. The code was compiled with Visual Studio 2005. [file 1748-7188-5-14-S1.ZIP › source_code/ANMM4CBR/NewCBR.plg]

```
# Build Log


### --------------------Configuration: NewCBR - Win32 Release--------------------


### Command Lines

Creating temporary file "C:\DOCUME~1\YAOBAN~1\LOCALS~1\Temp\RSP404.tmp" with contents
[
/nologo /MD /W3 /GX /O2 /D "WIN32" /D "NDEBUG" /D "_CONSOLE" /D "_MBCS" /D "_AFXDLL" /Fp"Release/NewCBR.pch" /YX /Fo"Release/" /Fd"Release/" /FD /c 
"E:\My_Curriculum\Bioinformatics\2007Spring\NewMultiCBR\CBR.CPP"
"E:\My_Curriculum\Bioinformatics\2007Spring\NewMultiCBR\main.cpp"
]
Creating command line "cl.exe @C:\DOCUME~1\YAOBAN~1\LOCALS~1\Temp\RSP404.tmp" 
Creating command line "link.exe /nologo /subsystem:console /incremental:no /pdb:"Release/NewCBR.pdb" /machine:I386 /out:"Release/NewCBR.exe"  .\Release\CBR.OBJ .\Release\DataHandle.obj .\Release\main.obj "

### Output Window

Compiling...
CBR.CPP
main.cpp
Linking...

### Results

NewCBR.exe - 0 error(s), 0 warning(s)
```
